# Predictive Parameters for Clinical Outcome in Patients with Critical Limb Ischemia Who Underwent Percutaneous Transluminal Angioplasty (PTA): A Systematic Review

**DOI:** 10.1007/s00270-017-1796-9

**Published:** 2017-09-18

**Authors:** Sanne M. Schreuder, Yvette M.G.A. Hendrix, Jim A. Reekers, Shandra Bipat

**Affiliations:** Department of Radiology and Nuclear Medicine, Academic Medical Center, University of Amsterdam, Meibergdreef 9, 1105 AZ Amsterdam, The Netherlands

**Keywords:** CLI, PTA, Amputation free survival, Survival

## Abstract

**Purpose:**

To identify possible risk factors in predicting clinical outcome in critical limb ischemia (CLI) patients undergoing percutaneous transluminal angioplasty (PTA).

**Materials and Methods:**

PubMed and EMBASE were searched for studies analyzing CLI and clinical outcome after PTA from January 2006 to April 2017. Outcome measures were ulcer healing, amputation free survival (AFS)/limb salvage and overall survival. Data on predictive factors for ulcer healing, AFS/limb salvage and survival were extracted.

**Results:**

Ten articles with a total of 2448 patients were included, all cohorts and based on prospective-designed databases. For ulcers, it seems that complete healing can be achieved in most of the patients within 1 year. No significant predictive factors were found. *AFS/limb salvage:* AFS rates for 1, 2 and 3 years ranged from 49.5 to 75.2%, 37 to 58% and 22 to 59%, respectively. Limb salvage rates for 1, 2 and 3 years ranged from 71 to 95%, 54 to 93.3% and 32 to 92.7%, respectively. All studies had different univariate and multivariate outcomes for predictive factors; however, age and diabetes were significant predictors in at least three studies. *Survival:* Survival rates for 1, 2 and 3 years ranged from 65.4 to 91.5%, 45.7 to 76% and 37.3 to 83.1%, respectively. Different predictive factors were found; however, age was found in 2 out of 5 studies reporting on predictive factors.

**Conclusions:**

In several studies two factors, age and diabetes, were found as predictive factors for AFS/limb salvage and survival in patients with CLI undergoing PTA. Therefore, we believe that these factors should be taken into account in future research.

**Level of Evidence:**

Level 2a.

## Introduction

Critical limb ischemia (CLI) due to peripheral arterial disease is a condition in which the lower extremity is threatened and is defined by ischemic rest pain, with or without ischemic tissue loss [1]. CLI has a great impact on healthcare and associated healthcare budget [2]. A number of risk factors are known to be associated with the development of CLI, which are diabetes mellitus, smoking, increased age, lipid abnormalities and low ankle-brachial pressure index [2].

Of the CLI patients, 10–40% will lose their leg within 6 months and the 1-year mortality rate is 25% in CLI patients who are not able to be revascularized [2–4].

Percutaneous transluminal angioplasty (PTA), with or without stenting, is an alternative approach to surgical bypass as a revascularization method in patients with CLI [5, 6]. Compared to surgery, it involves advantages such as minimal access trauma and shorter hospital stay. Therefore, PTA is more suited and often suggested as first-line therapy for high-risk CLI patients with a lower life expectancy [7–10].

To identify the effect of PTA, clinical outcomes such as wound healing, amputation free survival (AFS) and survival during follow-up are recorded and presented [11–17]. However, interpreting these clinical outcomes in this patient group is difficult, because of its heterogeneity in the risk factors such as comorbid diabetes, difference in age, renal failure or lifestyle factors such as smoking and obesity. We often see a discrepancy between a good revascularization result of the PTA, identified on digital subtraction angiography (DSA) and an unexpected poor clinical outcome with early amputation [9, 18, 19]. For future analysis of study results concerning endovascular treatment in CLI patients, it is important to identity which risk factors are associated with poor outcome.

Therefore, the aim of this systematic review was to identify risk factors in predicting poor clinical outcome in patients with CLI undergoing PTA with or without stenting. Drug eluting technologies were not included in the review to try to maintain homogeneity in the study population.

## Materials and Methods

This review was conducted according to the preferred reporting items for systematic review and meta-analysis (PRISMA) guidelines [20]. The review protocol was not published or registered in advance.

### Search Strategy

An electronic search was performed in the databases PubMed and EMBASE for studies analyzing CLI and clinical outcome after percutaneous revascularization. The search period was from January 2006 to April 2017. Search terms used for PubMed and EMBASE are listed below.

*PubMed* **“**Critical limb ischemia OR critical limb ischemia AND (angioplasty OR endovascular revascularization OR percutaneous intentional extraluminal revascularization OR subintimal OR endovascular therapy) AND (major amputation OR amputation free survival OR death OR ulcer healing OR wound healing OR mortality OR survival) AND Humans”.

*Embase*: (Critical limb ischamia OR critical limb ischemia) AND (percutaneous transluminal angioplasty balloon OR percutaneous transluminal angioplasty OR angioplasty OR stent OR revascularization) AND mortality OR (amputation OR major amputation OR leg amputation) OR (ulcer healing OR wound healing) OR (survival).

### Study Selection

#### Step 1

All retrieved articles were checked on title and abstract by one observer (X2). Duplicates, reviews, guidelines, comments, letters to the editor, conferences, case reports, study protocol and articles not containing CLI were excluded.

#### Step 2

All remaining articles were also checked on abstract by the same observer (X2). When studies contained less than fifty patients, patients did not receive PTA, the study was retrospective (we considered prospective database as prospective study) or the follow-up period was less than 1 year, these studies were excluded. To avoid exclusion of relevant articles, ambiguous articles were retrieved as full text and treated as potentially eligible articles. The observer double-checked step 2 and was not blinded to author and journal names.

### Inclusion of Relevant Articles

Three observers (X1, X2 and X3) independently checked all remaining articles for inclusion and exclusion criteria. Two observers (X1 and X2) each checked half of the relevant articles, and the findings were discussed with observer 3 (X3) who has experience on data extraction of 25 meta-analyses.

The inclusion criteria were as follows: (1) prospective study or prospective database (we considered prospective database as prospective study, hospital billing and other registries as retrospective); (2) patients with CLI as defined by Fontaine class III–IV or Rutherford class IV–VI (rest pain, non-healing ulcer or gangrene); (3) patients underwent (regular) PTA (no drug eluting stents); (4) >50 patients with CLI undergoing PTA; (5) data on outcome were available for at least 1 year of follow-up (outcomes were healing, AFS (major of minor) and overall survival); (6) separate data on CLI and PTA were available (in studies that included a variety of patients or treatments, for example data on CLI patient who underwent PTA or bypass surgery); and (7) finally, data on predictive factors were reported. Exclusion criterion was duplicate data.

### Data Extraction

Two reviewers (X1, radiologist with experience in extracting data of two reviews and X2, medical student) used a standardized form to extract data independently on study design characteristics, patient selection, baseline patient characteristics, procedure description, angiographic outcomes and complications, follow-up and dropout patients, clinical outcomes and predictive factors. Again, each observer extracted data of half of the articles and were double-checked by the third reviewer with experience on data extraction of 25 meta-analyses.

*Study design characteristics* The following data on study design characteristics were extracted: (1) study type (cohort, part of RCT or other); (2) study design (single center or multicenter and prospective study or prospective database retrospectively analyzed); (3) setting initiation institute (academic, tertiary or other); (4) department initiation by first author (radiology, surgery or other); (5) period of recruitment; (6) institutional review board approval (approved and informed consent obtained/waived, not approved or unclear); and (7) funding or a potential role of funders in the study (conflict of interest).

*Patient selection* The following data on patient selection were retrieved: (1) consecutive sample of patients enrolled (yes or no); (2) inclusion and exclusion criteria defined; and (3) spectrum of patients representative for CLI patients normally receiving PTA.

*Baseline patient characteristics* There were no age limits applied regarding patients. The following data on patient population were extracted: (1) number of patients included in the study and (2) analyzed in the final analysis; (3) age of patients (mean ± SD, median and/or range); (4) male-to-female ratio; (5) smoking (*n* + percentage); (6) diabetes mellitus (*n* + percentage); (7) hypertension (*n* + percentage); (8) dyslipidaemia (*n* + percentage); (9) renal failure (*n* + percentage); (10) coronary artery disease (*n* + percentage); (11) stroke history (*n* + percentage); (12) BMI < 18,5 kg/m2 (*n* + percentage); (13) other factors (*n* + percentage); (14) other baseline characteristics such as ankle-brachial index (ABI), toe pressure (mean ± SD in mmHg), ankle pressure, TcPO2 (mean ± SD in mmHg), ulcer classification (*n* + percentage), Fontaine classification (III or IV), Rutherford classification (IV, V and VI) and other characteristics when cited; and (15) anticoagulation/antiplatelet medication at baseline (*n* + percentage).

*Procedure description* The following data were extracted: (1) who performed the procedure (interventional radiologist, vascular surgeon or other); (2) experience defined (number of procedures performed or years of experience); (3) which procedure was performed (only PTA (balloon), PTA + stent placement or other); and (4) if the study was described in sufficient detail to permit its replication (if information was provided as stated in previous items 1–3).

*Angiographic outcomes and complications* data were extracted on how articles defined (1) technical success; (2) partial success/failure; (3) complete technical failure; (4) major complications; and (5) minor complications and how many successes, failures and complications occurred.

*Follow-up and dropout patients* The following data were extracted regarding follow-up: (1) a summary of follow-up time and scheme; (2) if all patients underwent the same follow-up (yes or no) and (3) were dropout patients adequately reported (yes or no, with or without reasons for dropout or unclear).

*Clinical outcomes and predictive factors* Data were extracted on the three previously defined outcome variables: (1) ulcer healing; (2) AFS (major of minor) or limb salvage and (3) overall survival at baseline and at least 1-year follow-up with a maximum of 5-year follow-up. Data on predictive factors either in terms of regression analysis (univariate or multivariate) were extracted.

### Data Analysis

All data at baseline were presented as number plus percentage, with the exception of age, which is presented as a mean. Because standard deviation was not available in all datasets, result on baseline could not be pooled.

Data on ulcer healing, AFS and overall survival at baseline and at least 1-year follow-up were recorded. Data on predictive factors for ulcer healing, AFS (also limb salvage) and survival were extracted as reported in papers. As anticipated, the number of studies was limited. The data were heterogeneously presented so even meta-analysis with random effect approach would not be suitable for pooling predictive values. All data are therefore presented per study.

## Results

### Search, Selection and Inclusion of Relevant Articles

The search yielded 1635 studies: 734 from Pubmed and 901 from EMBASE (see Appendix 1).

After excluding duplicates (240), letters/comments/editorials (57), conferences (354), case reports (42), other languages than English, Dutch, French or German (38), reviews and guidelines (228), study protocols (7), articles not involving CLI (37) and seven articles of which the full article could not be obtained, 625 articles on CLI remained.

**Fig. 1:**
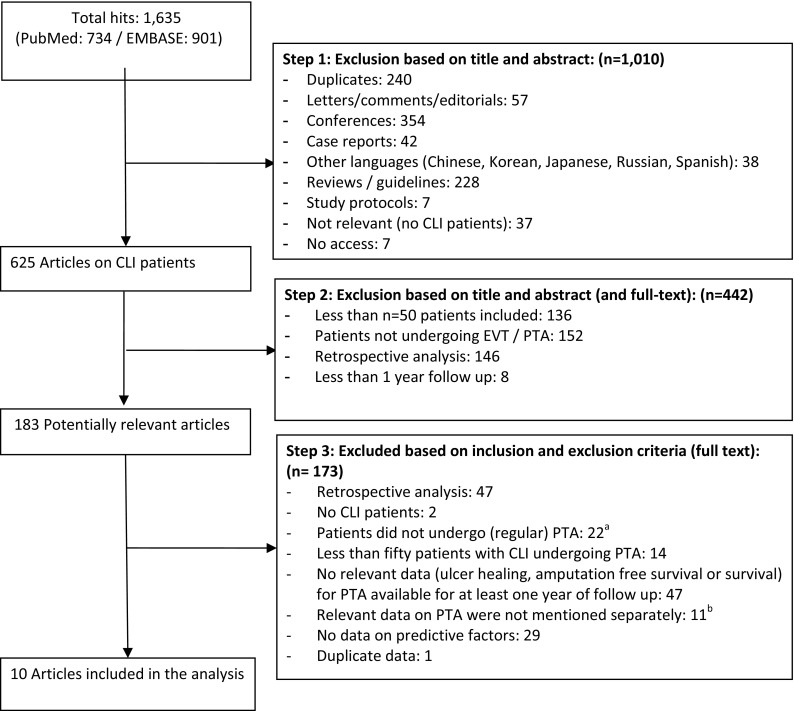
Search, selection and inclusion of relevant articles. ^a^Patients did not undergo primary or standard PTA (e.g., use of primary stenting or drug eluting stent) or it was not clear what number of patients did undergo PTA. ^b^In several studies patients did undergo PTA; however, no data were separately mentioned from other procedures (e.g., bypass surgery)

Subsequently, articles were excluded based on title and abstract because they had less than 50 patients (136), they did not undergo PTA (152), were retrospective in nature (146) or had less than 1 year of follow-up (8) which yielded 183 potentially relevant articles. Full texts of these articles were checked on inclusion criteria: 173 articles did not meet the inclusion criteria and ten studies were included for data extraction (see Fig. 1) [21–30].

### Study Design Characteristics

Of the ten articles included, all were cohort studies; most studies were performed based on prospective-designed databases and were single center. In all studies, there was no role of funders (see Table 1).

**Table 1:** Study design characteristics

References	Type of study	Data collection	Study design^a^	Initiation institute, department	Recruitment period	Institutional review board approval	Funding received	Funders role in study
[21]	Cohort	Prospective database	Multicenter	Surgery	Jan 2000–Dec 2007	Approved and requirement for IC waived	No	No
[22]	Cohort	Prospective	Single center	Radiology	Jul 2003–Dec 2007	Approved and IC obtained	Unclear	No
[23]	Cohort	Prospective database	Single center	Surgery	2007–2012	Approved	No	No
[24]	Cohort	Prospective	Single center	Surgery	Jan 1999–Jun 2004	Approved and IC obtained	No	No
[25]	Cohort	Prospective database	Single center	Surgery	Feb 2004–Feb 2012	Approved	No	No
[26]	Cohort	Prospective database	Single center	Surgery	Mar 2003–Sep 2010	Approved and IC obtained	No	No
[27]	Cohort	Prospective database	Multicenter	Cardiology	Apr 2004–Jun 2011	Unclear	No	No
[28]	Cohort	Prospective	Multicenter	Cardiovasular center	Dec 2009–Jul 2011	Approved and IC obtained	No	No
[29]	Cohort	Prospective	Single center	Radiology	Unclear	Approved and IC obtained	No	No
[30]	Cohort	Prospective database	Single center	Surgery	Apr 2010–Dec 2012	Unclear	No	No

^a^ We consider studies with authors from different centers as multicenter

### Patient Selection

The patient selection was consecutive in most of the studies. In all studies, patients were included with CLI; however, the spectrum of patients was equivocal, as in one study only patients > 80 years were included [21], only diabetic patients [22], only hemodialysis patients [27] or patients with Rutherford V and VI [28] (see Table 2).

**Table 2:** Patient selection criteria

References	Consecutive sample	Inclusion/exclusion criteria	Spectrum of patients representative
[21]	Consecutive	Inclusion Patients with CLI (ischemic rest pain or tissue loss: ulceration or gangrene) Patients aged at least 80 years Patient who underwent PTA	No, only patients >80 years
[22]	Consecutive	Inclusion Diabetic patients with Fontaine stage IV CLI, not suitable for surgical recanalization Patients undergoing infrainguinal subintimal angioplasty	No (only diabetic patients and stage IV CLI)
[23]	Unclear	Inclusion Patients with CLI who underwent isolated intervention for tissue loss (Rutherford V and VI) Patient with end stage renal disease on hemodialysis compared to patients without ESRD (no-hemodialysis)	No
[24]	Consecutive	Inclusion All patients presenting with chronic CLI Definition of CLI: (1) presence of ischemic rest pain for >2 weeks or ischemic tissue loss associated with (2) an absolute ankle pressure of <50 mm Hg or great toe pressure of <30 mm Hg Exclusion: Patients with acute limb ischemia	Yes
[25]	Consecutive	Inclusion Patients undergoing an attempt at infrapopliteal angioplasty for CLI or bypass graft outflow vessel stenosis	Yes
[26]	Consecutive (stated as ‘all patients’)	Inclusion All patients who underwent endovascular therapy for crural arteries (defined as arteries below the popliteal segment) Chronic CLI, defined as >2 weeks of rest pain, ulcers, or tissue loss, attributed to arterial occlusive disease	Yes
[27]	Consecutive	Inclusion Patients with hemodialysis who have CLI with ischemic wounds, who underwent EVT for isolated infrapopliteal lesions Exclusion Patients with CLI who underwent multilevel EVT due to tibial artery lesions combined with femoropopliteal (FP) lesions or aorto-iliac FP lesions CLI patients with functionally unsalvageable limbs with ischemic ulcer or gangrene spreading extensively past the ankle Patients with functional contraindications, including those bedridden without intractable ischemic pain Patients with psychiatric contraindications, including those with dementia or mental retardation from whom understanding of the treatment cannot be gained Patients with social contraindications for whom continuation of treatment would be difficult due to lack of cooperation from family members or nurses	No (only hemodialysis patients)
[28]	Consecutive	Inclusion Patients with tissue loss (Rutherford class V or VI) caused by infrainguinal disease Available postprocedural skin perfusion pressure (SPP) and ankle-brachial index Clinical outcomes including 12-month AFS, freedom from major adverse events, defined as major amputation or any reintervention and complete wound healing Exclusion Previous major amputation Unsalvageable limb defined as extensive ischemic ulceration or gangrene beyond the transmetatarsal level that would eventually require major amputation after EVT Concurrent iliac artery disease CLI attributable to acute arterial occlusion or to non-atherosclerotic or inflammatory diseases CLI presenting with rest pain and no tissue loss (Rutherford IV)	No (only Rutherford V and VI)
[29]	Unclear	Inclusion CLI symptoms (Rutherford categories IV–VI) DSA documentation of infrapopliteal obstructive arterial disease Bail-out stenting after suboptimal and/or complicated below-knee angioplasty Reference diameter of native tibial vessel less than 4 mm Exclusion History of severe contrast allergy/hypersensitivity Hypersensitivity to aspirin and/or clopidogrel Systemic coagulopathy or hypercoagulation disorders Acute limb ischemia Buerger disease Deep vein thrombosis Bifurcation and/or trifurcation lesions Previous use of other drug eluting stent (not SES) Stenting indications after suboptimal and/or complicated balloon angioplasty Elastic recoil Flow-limiting dissection Residual stenosis more than 30%	Yes
[30]	Consecutive	Inclusion All patients with CLI who were not eligible for BTK reconstructive vascular surgery	Yes

### Baseline Patient Characteristics

In total, 2448 patients were included who were CLI patients and underwent PTA with or without bare metal stent placement. Mean ages ranged from 50 to 85.9 years. Male-to-female ratio was 816:534 in the seven studies mentioning this ratio [21–23, 27–30]. In addition, a broad range of risk factors was present: smoking rate from 6.9 to 58.3%, diabetes from 49.1 to 100%, hypertension from 51.6 to 98%, dyslipidaemia from 21.1 to 65% and renal disease up to 100%. Other risk factors such as coronary artery disease, cerebrovascular disease and stroke were also present in the majority of patients (see Table 3).

**Table 3:** Patient baseline characteristics: risk factors

References	N of patient analyzed	Age (years) Mean ± SD; median + range	Male: female	Smoking *N* (%)	Diabetes mellitus *N* (%)	Hypertension *N* (%)	Dyslipidaemia *N* (%)	Renal failure *N* (%)	Coronary artery disease *N* (%)
[21]	277 CLI patients who underwent PTA	85.9 ± 4.0	77:200	19 (6.9%)	143 (51.6%)	212 (51.6%)	75 (27.1%)	58 ± 21	196 (70.8%)
							hyperlipidaemia	eGFR mean ± SD	
[22]	60	69.4 ± 9.4	41:19	35 (58.3%)	60 (100%)				25 (41.7%)
		Range 49–86			Duration: 21.9 ± 12 years				Cardiac disease
[23] Non- hemodialysis group	164	50 ± 13	82:82	21 (13%)	126 (77%)	152 (93%)	96 (59%)		65 (40%)
[23] Hemodialysis group	78	66 ± 12	44:34	15 (20%)	68 (88%)	76 (98%)	51 (65%)	78 (100%)	33 (43%)
[24]	207 patients who underwent PTA	77.1 ± 9.7			119 (57.5%)				
[25]	459 limbs in 413 patients	71 ± 12	271:188 (limbs)	203 (58%)	342 limbs (75%)	386 (84%)	279 (61%)	71 (15%) Dialysis dependent	229 (50%)
		Range 31–96						Serum creat >2: 102 (22%)	
[26]	527 limbs in 478 patients	73.9 ± 0.53	315:212 (limbs)	62 (12%)	256 limbs (49.1%)	344 limbs (70%)		Dialysis dependent limbs: 38 (7.4%)	228 limbs (45%)
								Creat >150: 39 (8%)	
[27] Minor tissue loss group	340 patients with minor tissue loss	69.2 ± 9.6	265:75	112 (32.9%)	260 (76.5%)	244 (71.8%)	79 (23.2%)	340 (100%)	198 (58.2%)
[27] Major tissue loss group	109 with major tissue loss	66.5 ± 10.4	85:24	47 (43.1%)	82 (75.2%)	83 (76.1%)	23 (21.1%)	109 (100%)	63 (57.8%)
[28]	211	73.6 ± 9.7	134:77	Past 87 (41%)	152 (72%)	166 (79%)	72 (34%)	129 (62%)	100 (47%)
								Dialysis: 111 (53%)	
				Current 19 (9%)					
[29]	41 (only bare metal stent (BMS))	71.55 ± 8.27	37:4	21 (51.2%)	31 (75.6%)	32 (78.0%)	28 (68.3%)	17 (41.5%) Renal disease	19 (46.3%)
					Insulin dependent 14 (45.2%)		Hyperlipidaemia		cardiac disease
[30]	70	72 Range 43–93	51:19	38 (54%) history of smoking	50 (71%)	44 (63%)			38 (40%) cardiac disease

References	Stroke history *N* (%)	Other risk factors *N* (%)
[21]	61 (22.0%)	Pulmonary disease: 46 (16.6%)
	Cerebrovascular disease	
[22]	15 (25%)	Cholesterol: 161.4 ± 25.5 mg/dl (range 81–246)
	Cerebrovascular disease	Creatinine: 1.3 ± 1.1 mg/dl (range 0.5–7.5)
		Previous peripheral intervention: 12 (20%)
		Retinopathy: 35 (58.3%)
[23] Non- hemodialysis group	36 (22%)	Metabolic syndrome: 110 (67%)
	Cerebrovascular disease	Hypothyroidism: 26 (16%)
[23] Hemodialysis group	19 (24%)	Metabolic syndrome: 46 (59%)
		Hypothyroidism: 10 (13%)
[24]		
[25]	74 (16%)	Congestive heart failure: 120 (26%)
		COPD: 37 (8%)
[26]	69 limbs (14%)	>80 years 225 limbs (38.33%)
[27] Minor tissue loss group	88 (25.9%)	Hemodialysis: 340 (100%)
	Cerebrovascular disease	CRP > 5.0 mg/dl: 56 (16.6%)
		COPD: 26 (7.7%)
[27] Major tissue loss group	31 (28.4%)	Hemodialysis: 109 (100%)
		CRP > 5.0 mg/dl: 37 (33.9%)
		COPD: 12 (6.8%)
[28]	47 (22%)	BMI < 18.5%: 22.0 ± 3.4 (*n* = 207)
		Serum albumin: 3.6 ± 0.5 (n-197)
		HbA1C: 6.2 ± 1.2 (*n* = 198)
		Anemia: 150 (71%)
		Heart Failure: 62 (30%)
		Previous myocardial infarction: 37 (18%)
[29]		
[30]		COPD: 10 (14%)

ABI was mentioned in only small number of studies, other measurements such as toe pressure and ankle pressure were only mentioned in the study of Strom et al. (toe pressure mean 30 mmHg [range 0–60 mmHg] and ankle pressure mean 50 mmHg [range 0–60 mmHg]) [30]. The TcPO2 was not mentioned in any of the studies. The disease severity in terms of Fontaine classification or Rutherford category was described heterogeneously (see Table 4).

**Table 4:** Patient baseline characteristics: risk factors, continuing Table 3

References	Ankle-brachial index (ABI) Mean ± SD; median + range	Fontaine classification, Rutherford category or other classification	Anticoagulation/antiplatelet medication at baseline (N and percentage)
[21]	NA	Fontaine III: 47 (17%)	NA
		Fontaine IV: 230 (83%)	
[22]		Fontaine IV: 60 (100%)	NA
		TASC B: 9 (15%)	
		TASC C: 24 (40%)	
		TASC D: 27 (45%)	
[23] Non-hemodialysis group		Rutherford V: 139 (85%)	Aspirin and Heparin: 164 (100%)
		Rutherford VI: 25 (15%)	
[23] Hemodialysis group		Rutherford V: 48 (62%)	Aspirin and Heparin: 78 (100%)
		Rutherford VI: 30 (38%)	
[24]	0.45 (0.15–1.47)	Rutherford IV: 30 (14.5%)	
		Rutherford V: 175 (84.5%)	
		Rutherford VI: 2 (1%)	
[25]		Tissue loss 363 (79%)	Aspirin: 63
		Rest pain 57 (12%)	Clopidogrel: 32
			Warfarin: 20
		Acute limb ischemia 10 (3%)	
		Threatened graft 28 (6%)	
		TASC A 75 (16%)	
		TASC B 101 (22%)	
		TASC C 126 (27%)	
		TASC D 157 (34%)	
[26]		Rutherford IV: 158 limbs (30%)	
		Rutherford V and VI: 358 limbs (67.9%)	
[27]	0.57 ± 0.24	Ulcer classification: infected 119 (35.0%)	All patients Aspirin 100 mg/day and Clopidogrel 75 mg/day. Cilostazol 200 mg/day at and after procedure
[27]	0.59 ± 0.21	Ulcer classification: infected 74 (67.9%)	All patient Aspirin 100 mg/day and Clopidogrel 75 mg/day. Cilostazol 200 mg/day at and after procedure
[28]	0.72 ± 0.23 (*n* = 180)	Ulcer classification: wound infection 34 (16%)	Aspirin: 184 (87%)
		Rutherford V: 173 (82%)	Cilostazol: 107 (51%)
			Clopidogrel: 94 (45%)
		Rutherford VI: 38 (18%)	
[29]		Fontaine III/Rutherford IV: 15 (36.6%)	All patients Aspirin 100 mg/day and Clopidogrel 75 mg/day 3 days before procedure
		Fontaine III/Rutherford V: 16 (39.0%)	
		Fontaine IV/Rutherford VI: 10 (24.4%)	
[30]	NA	Ulcer classification: ischemic ulcers 59 (84%)	All patients Acetylsalicylic acid (ASA) 75 mg daily after the procedure
			Clopidogrel postoperatively in selected cases (n = 4)

### Procedure Description, Outcomes and Complications

In most studies, it was not clear who performed the procedure. Moreover, the experience of the operator was not defined in any of the studies. In none of the studies, the procedure was described in sufficient detail to replicate. The angiographic outcome in terms of technical success was defined well, and complications were reported in detail.

All data on procedure description and outcomes are given in detail in Table 5.

**Table 5:** Description of angiographic procedures, angiographic outcomes and complication

References	Description of angiography				Angiographic outcomes and complications	
	Who performed procedure	Experience	Type of procedure	Procedure description in sufficient detail to replicate	Definitions outcomes (definitions and number)	Complications (definitions and number)
[21]	Unclear	Unclear	-PTA -Stent placement in case of dissection or a long lesion	No	NA	NA
[22]	Interventional radiologist	Unclear	-PTA	No	-Technical success: visualization of a correctly dilated subintimal lumen, with adequate run-in and run-off vessels, without immediate complications: 91.7% (55/60)	-Procedure: 1 dissection treated by stenting, 1 hematoma at re-entry site, 1 pseudoaneurysm, 1 retroperitoneal hematoma; all treated conservatively -Peri-procedural mortality 5% (3 patients): myocardial infarction in 2 cases and renal failure in 1 patient
[23] Non-hemodialysis group	Unclear	Unclear	-PTA -Stent placement primarily or in case of flow-limiting dissections, intimal flaps or poor technical results -Atherectomy (Limited number)	No	-Technical success: a patient target tibial vessel with successful revascularization of the intended angiosome or inline flow across the ankle into the foot -Technical failure: 4% (6/164)	-Major complication was defined as any event, regardless of how minimal, not routinely observed after endoluminal therapy that required treatment with a therapeutic intervention or rehospitalisation within 30 days of procedure. Systemic complications were sepsis, related to cardiac, pulmonary or renal system. Local complications were related to access site, surgical wounds and the treated limb: 1% (1/164) systemic and 0% (0/164) local complications -Lesion complications (site of intervention): 2% (3/164) -Death < 30 days of procedure was considered procedure-related and a perioperative death: 0%
[23] Hemodialysis group	Unclear	Unclear	-PTA -Stent placement primarily or in case of flow-limiting dissections, intimal flaps or poor technical results -Atherectomy (Limited number)	No	-Technical success: a patent target tibial vessel with successful revascularization of the intended angiosome or inline flow across the ankle into the foot -Technical failure: 2% (2/78)	-Major complication was defined as any event, regardless of how minimal, not routinely observed after endoluminal therapy that required treatment with a therapeutic intervention or rehospitalisation within 30 days of procedure. Systemic complications were sepsis, related to cardiac, pulmonary or renal system. Local complications were related to access site, surgical wounds and the treated limb: 4% (3/78) systemic and 1% (1/78) local complications -Lesion complications (site of intervention): 10% (8/78) -Death < 30 days of procedure was considered procedure-related and a perioperative death: 2% (*n* = 2)
[24]	Unclear	Unclear	-PTA (with or without stenting)	No	-Primary technical success 196/207 (94.7%) -Repeat target extremity revascularizations: re-PTA in 54/207 limbs, reconstructive surgery in 26/207 limbs	NA
[25]	Unclear	Unclear	-PTA (with or without stent placement) -Atherectomy in 6 patients	No	-Technical success defined as a residual stenosis <30%: 427/459 (93%)	Intraprocedural complications: flow-limiting dissections 69 (15%), vessel spasm 29 (6%), arteriovenous fistulas 6 (1%), distal embolization 17 (4%), rupture 1 (0.2%) Postoperative complications: access site arterial injury 20 (4%), acute kidney injury 11 (2%), acute myocardial infarction 4 (1%), congestive heart failure 4 (1%), dysrhythmia 5 (1%), respiratory failure or pneumonia 5 (1%), gastro-intestinal bleed/hematemesis 5 (1%), cerebrovascular accident 3 (1%) In-hospital mortality: 11 patients (2%) 30-day mortality: 26 (6%)
[26]	Interventional radiologist	Unclear	-PTA	No	Successful if direct flow was restored in the treated vessel with less than 30% residual stenosis: number NA	Complications: embolus 17 (2.9%), groin hematoma 16 (2.7%), target vessel thrombosis 14 (2.4%), vessel perforation 9 (1.5%), vessel rupture 7 (1.2%), deterioration in ischemia 2 (0.3%), flow-limiting dissection 1 (0.2%), arteriovenous fistula 1 (0.2%), retroperitoneal hemorrhage 1 (0.2%), others 8 (1.4%)
[27] (minor tissue loss group and major tissue loss group)	Cardiovascular interventionalist or vascular surgeon	Unclear	-PTA	No	Technical success was defined as achieving a degree of residual stenosis < 30% at the target lesion site and achieving straight-line flow from the aorta down to either a patent dorsalis pedis or plantar artery: 241/340 (70.9%) in minor tissue loss group. 77/109 (70.6%) in major tissue loss group (*p* = 0.961)	Perioperative mortality 9/340 (2.6%) in minor tissue loss group; 3/109 (2.8%) in major tissue loss group
[28]	Unclear	Unclear	-Below the knee: PTA (plain angioplasty or cutting balloon) -Femoral lesions: PTA (plain angioplasty or cutting balloon) or nitinol stent placement	No	Technical success was defined as straight flow to the foot: 197/211 (93%)	NA
[29] (only the bare metal stent group)	Unclear	Unclear	-BMS placement	No	Technical success was defined as recanalization of at least one straight-line of blood flow to the distal foot: 93.6%	-Retroperitoneal hemorrhage: 1, self-limiting -30-day mortality rate: 1
[30]	Vascular surgeon	Unclear	-PTA (with or without stent placement)	No	NA	One patient (2%) developed a groin hematoma demanding surgical evacuation One patient (2%) presented with acute abdomen and respiratory distress suspected of acute mesenteric ischemia Two patients died within 30 days (perioperative mortality; 3%) due to toxicity awaiting amputation (*n* = 1) and cerebral hemorrhage occurring after a minor amputation (n = 1)

### Follow-Up and Dropout Patients

The follow-up was not homogeneous, but in general 1 month, 3-, 6- and 12-month follow-up was done. Patients did not undergo the same follow-up in seven studies, while in three studies patients did undergo the same follow-up. Dropout rates are poorly reported. Only one study [24] accurately reported dropouts, with missing baseline information as most frequent reason for dropout. Follow-up ranged from less than 1 month up to 109 months. All details are given in Table 6.

**Table 6:** Follow-up and dropouts of patients

References	Summarize follow-up time and scheme	Undergo same follow-up	Dropouts reported
[21]	1, 6 and 12 months and annually thereafter	No: mean 2,0 years	Study registry, dropouts not reported
[22]	Not stated	No: range 1–48 months, 22.8 ± 14.9, median 22.5 months	None
[23] Non-hemodialysis and hemodialysis group	1, 3 and every 6 months following their procedure	No: means or ranges stated	Study registry, dropouts not reported
[24]	2, 6 and 12 months	Yes	Yes (missing baseline information (10), refusal to undergo vascular imaging (2), withdrawal of informed consent (1), lack of follow-up data (5)
[25]	2 weeks, then every 3 months for 1 year and every 6 months thereafter	No: average 15 months (range 0–85 months)	Early deaths reported. Dropouts in further follow-up are not stated
[26]	1, 6, 12, 36 months	No: mean 26.9 ± 0.54 months, median 40 months with a maximum of 109 months	No
[26]	1, 3, 6 months and every 3 months thereafter up to 3 years	Yes	None
[28]	1, 3, 6, 12 months	Yes	None
[29]	1, 3, 6, 12 months and yearly thereafter	No: mean 17.15 months ± 1.73, range 0.7–36 months	No
[30]	6 weeks and 1 year (no standard FU after 1 year)	No: median 20 months (range 0–41 months)	None

### Clinical Outcomes: Ulcer Healing, AFS/Limb Salvage and Survival

#### Ulcer Healing

In three studies [22, 25, 28], data on ulcer healing were given. It seems that complete healing can be achieved in most of the patients within 1 year [25, 28]. Details are given in Table 7.

**Table 7:** Follow-up data on ulcer healing

References	6 months	1 year	3 year
[22]			Healing 45 (75%)
			Improved 7 (11.6%)
			Stable 4 (6.7%) (Data at latest FU, however, FU ranges from 1 to 48 months)
[25]	*N* = 361	*N* = 192	
	Complete healing 15%	Complete healing 63%	
		Improved 30%	
	Improved 55%		
	Stable 27%	Stable 8%	
	Worse 2%	Worse 0.5%	
[28]		*N* = 164	
		87%	

### AFS or Limb Salvage

In all studies [21–30], data on AFS or limb salvage were given. One-year AFS ranged from 49.5 to 75.2%, 2-year AFS from 37 to 58% and 3-year AFS from 22 to 59%. The limb salvage rates for 1 month, 1 year, 2 year and 3 year range from 95 to 97.4%, 71 to 95%, 54 to 93.3% and 32 to 92.7%, respectively. All data are given in Table 8.

**Table 8:** Follow-up data on AFS or limb salvage

References	1 month	1 year	2 years	3 years	4 years	5 years
AFS (Amputation free survival)						
[21]	93.1%	62.4%	53.0%	44.3%	35.3%	32.9%
[23] Non-hemodialysis group				54 ± 4%		
[23] Hemodialysis group				22 ± 9%		
[26]		75.2%		59.0%		
[26] Minor tissue loss group		63.5 ± 2.9%	51.0 ± 3.3%	44.1 ± 3.7%		
[26] Major tissue loss group		49.5 ± 5.5%	37.0 ± 6.1%	29.1 ± 7.0%		
[28]		73.9%				
[30]		68%	58%			
Limb salvage						
[21]	97.4%	88.8%	85.4%	82.6%	80.2%	78.3%
[22]	95% (3 patients, 5% above knee amputation)	95%	93.3%			
[23] Non-hemodialysis group		76 ± 3%	74 ± 4%	69 ± 4%		
[23] Hemodialysis group		71 ± 5%	54 ± 7%	32 ± 8%		
[24]	96.5%	81%				
[25]	96%	84%				81%
[26]				92.7%		
[26] Minor tissue loss group		87.4 ± 1.8%	84.4 ± 2.1%	83.7 ± 2.2%		
[26] Major tissue loss group		73.9 ± 4.3%	71.2 ± 4.5%	71.2 ± 4.5%		
[29]				80.3%		

#### Survival

Survival rates were described in nine studies [21–29] with at least 3-year follow-up in most of the studies (see Table 9). The survival rates for 1 month, 1 year, 2 years and 3 years range from 94 to 100%, 65.4 to 91.5%, 45.7 to 76% and 37.3 to 83.1%, respectively.

**Table 9:** Follow-up data on survival

References	1 month	1 year	2 years	3 years	4 years	5 years
[21]	94.9%	66.7%	57.7%	50.4%	42.3%	39.9%
[22]	95%	91.5%		83.1%		
[23] Non-hemodialysis group	100%	83 ± 3%	76 ± 3%	67 ± 4%		
[23] Hemodialysis group	98%	70 ± 6%	53 ± 7%	45 ± 8%		
[24]	94%	70.6%				
[25]		83%		64%		49%
[26]	97.2%	82.9%		62.4%		
[26] Minor tissue loss group		74.9 ± 2.6%	63.7 ± 3.2%	54.0 ± 3.7%		
[26] Major tissue loss group		65.4 ± 5.2%	45.7 ± 6.4%	37.3 ± 7.7%		
[28]		80.6%				
[29]				70.7%		
[30] Non-amputated group	97%	81%				
[30] Amputated group		64%				

### Predictive Factors

When data were available on predictive values, these data were also extracted (see Table 10). However, these data were presented heterogeneously. We extracted all data as given in the studies. In general for univariate analysis, data were given either (1) at a time point (e.g., AFS at 2 years) by Fisher exact test or Chi-square test (2 × 2 tables) or Student’s *t* test (continuous normally distributed data) or Mann–Whitney tests (continuous not normally distributed data) or by association tests (continuous data) or (2) as time dependent by Kaplan–Meier analysis (with log rank test, for binary data) or Cox regression analysis (for multinomial or continuous data). Finally, multivariate analysis in either stepwise multiple regression analysis was used (at one time point) or Cox proportional regression analysis (for time dependent data) was performed.

**Table 10:** Prediction factors by outcome

References	Factors found to be significant in univariate analysis	Factors predictive (with p-values)
*Ulcer healing*		
[22]	Univariate analysis by fisher exact test, Chi-square test, Student’s t test (*p* < 0.05)	Stepwise multiple logistic regression
	Diabetes duration (*p* = 0.05)	HbA1c (*p* = 0.001)
	HbA1c (*p* = 0.002)	Serum creatinine levels (*p* = 0.03)
	Creatinine (*p* = 0.04)	
	Site of recent canalization (*p* = 0.03)	
[28]	Univariate analysis, logistic regression	Not available
	Skin perfusion pressure (*p* = 0.022)	
	Ankle-brachial Index (*p* > 0.05)	
*AFS or limb salvage*		
[21]^a^	Univariate analysis by fisher exact test, Chi-square, Mann–Whitney U test and Kaplan–Meier method (*p* < 0.05)	Cox regression with backward selection
AFS at 2 years	Age (*p* < 0.004)	AFS decreased for increased age, decreased EGR, diabetes, coronary artery disease and bypass surgery
	EGFR (*p* = 0.015)	
	Diabetes (*p* = 0.003)	
	Coronary artery disease (*p* = 0.004)	
	Foot gangrene (*p* = 0.025)	
	Level of vascularization (*p* = 0.004)	
	Technique of revascularization (*p* = 0.005)	
[23]	Univariate analysis in Kaplan–Meier and log rank or associations (*p* < 0.05)	Cox proportional regression analysis
Limb salvage (only hemodialysis group)	Improvements in hemodynamics after intervention (*p* = 0.02)	Improvements in hemodynamics after intervention (*p* = 0.009)
	Improvement in symptoms (*p* = 0.02)	Improvement in symptoms (*p* < 0.001)
[23] AFS (only hemodialysis group)	Univariate analysis in Kaplan–Meier and log rank or associations (*p* < 0.05)	Cox proportional regression analysis
	Presence of hyperlipidemia (*p* = 0.006)	MACE (*p* = 0.005)
	Cerebrovascular disease (*p* = 0.008)	Metabolic syndrome (*p* = 0.02)
	Diabetes (*p* < 0.001)	
	Metabolic syndrome (*p* < 0.001)	
	Modified cardiac risk (*p* = 0.02)	
	High-risk group (*p* = 0.04)	
	Presence of MACE (*p* = 0.02)	
	Elevated Finn score (*p* = 0.03)	
[24]	Not available	Cox proportional regression analysis
Limb salvage		Advanced age
		Diabetes
[25]	Univariate analysis using Kaplan–Meier and log rank (*p* < 0.05)	Cox proportional regression analysis
Limb salvage	TASC class (*p* = 0.006)	TASC class (*p* = 0.031)
	Not being a candidate for bypass (*p* < 0.001)	Not being a candidate for bypass (*p* < 0.001)
	Dialysis (*p* < 0.001)	
	Serum > 2.0 mg/dl (*p* = 0.02)	
[26]	Univariate analysis by Fisher exact test (*p* < 0.05)	Not available
AFS at 1 year	Coronary artery disease (*p* < 0.001)	
	Rutherford category (*p* < 0.001)	
	Renal disease (*p* = 0.030)	
[26]	Univariate analysis by Fisher exact test (*p* < 0.05)	Not available
AFS at 3 year	Age < 60 (*p* = 0.015)	
	Coronary artery disease (*p* < 0.001)	
	Rutherford category (*p* < 0.001)	
	Diabetes (*p* < 0.003)	
	Renal disease (*p* = 0.001)	
[26]	Univariate analysis by Fisher exact test (*p* < 0.05)	Not available
Limb salvage	Rutherford category (*p* = 0.016)	
	Diabetes (*P* = 0.020)	
[26]	Univariate analysis, Kaplan–Meier method and log rank (*p* < 0.05)	Cox proportional regression analysis
Major amputation (Minor tissue loss group)	Age < 60 (*p* = 0.003)	Age < 60 (*p* = 0.014)
	Nonambulatory (*p* = 0.036)	HbA1c ≥ 6.8% (*p* = 0.026)
	Hyperlipidemia (*p* = 0.027)	C-reactive protein > 5.0 mg/dl (*p* < 0.001)
	HbA1c ≥ 6.8% (*p* < 0.001)	Albumin < 3.0 g/dl (*p* = 0.007)
	C-reactive protein > 5.0 mg/dl (*p* < 0.001)	
	Albumin < 3.0 g/dl (*p* < 0.001)	
	Achieving technical success (*p* = 0.049)	
[26]	Univariate analysis, Kaplan–Meier method and log rank (*p* < 0.05)	Cox proportional regression analysis
Major amputation (Major tissue loss group)	Nonambulatory (*p* < 0.001)	Nonambulatory (*p* < 0.001)
	Heel location (*p* = 0.05)	Calcified lesions (*p* = 0.029)
	Calcified lesions (*p* = 0.048)	
[28]	Univariate analysis, logistic regression	Not available
AFS at 1 year	Skin perfusion pressure (*p* = 0.018)	
	Ankle-brachial index (*p* > 0.05)	
[29]^b^ Limb salvage	Not available	Cox proportional regression analysis
		No factors identified (tested)
[30] Overall amputation or major amputation	Univariate analysis by Fisher exact test (*p* < 0.05)	Not available
	None of the factors tested was significant	
*Surviva*l		
[22]	Univariate analysis by fisher exact test, Chi-square test, Student’s t test and Kaplan–Meier and log rank (*p* < 0.05)	Cox proportional regression analysis
Survival	Age (*p* = 0.002)	Age (*p* = 0.0001)
	Creatinine (*p* = 0.004)	
	Ulcer healing (*p* = 0.03)	Ulcer healing (*p* = 0.008)
[23]	Univariate analysis in Kaplan–Meier and log rank or associations (*p* < 0.05)	Cox proportional regression analysis
Survival (only hemodialysis group)	Cerebrovascular disease (*p* = 0.014)	Presence of MACE (*p* = 0.04)
	Diabetes (*p* = 0.003)	Major limb loss (*p* = 0.04)
	Presence of hyperlipidemia (*p* = 0.04)	
	Presence of MACE (*p* = 0.005)	
	Major limb loss (*p* = 0.008)	
[25]	Univariate analysis using Kaplan–Meier and log rank (*p* < 0.05)	Cox proportional regression analysis
Survival	Factors not given	Age 71–80 years (*p* = 0.042)
		Age > 80 (*p* < 0.001)
		Serum creat > 2.0 mg/dl (*p* = 0.038)
		Congestive heart failure (p = 0.04)
		Not being a candidate for bypass (p = 0.002)
[26]	Univariate analysis by Fisher exact test (*p* < 0.05)	Not available
Survival at 3 year	Age (*p* = 0.003)	
	Coronary artery disease (*p* < 0.001)	
	Rutherford category (*p* < 0.001)	
	Diabetes (*p* = 0.007)	
	Renal disease (*p* = 0.005)	
[29]**	Not available	Cox proportional regression analysis
Survival		No factors identified (tested)
[30]	Univariate analysis by Fisher exact test (*p* < 0.05)	Not available
(Death < 1 year)	None of the factors tested was significant	

^a^ In this study, although data (ulcer healing, AFS or survival) were reported separately for PTA, data of regression analysis was presented combined both groups: PTA and bypass surgery

^b^ In this study, although data (ulcer healing, AFS or survival) were reported separately for PTA with Bare Metal Stent (BMS), data of regression analysis was presented for both PTA with BMS and PTA with drug eluting stent. The cox regression showed no difference between both groups

**Table 11:** Details search strategy

Search terms		Number of hits
*PUBMED*		
#1	Search “Critical limb ischemia OR critical limb ischemia”	4246
#2	Search (angioplasty OR endovascular revascularization OR percutaneous intentional extraluminal revascularization OR subintimal OR endovascular therapy)	95,820
#3	Search (major amputation OR amputation free survival OR death OR ulcer healing OR wound healing OR mortality OR survival)	2,061,511
#4	Search (#1 AND #2 AND #3)	915
#5	Search (#1 AND #2 AND #3) Sort by: Relevance Filters: published between January 2006 and April 2017; Humans	734
*EMBASE*		
#1	critical limb ischemia.mp. OR *critical limb ischemia	2669
#2	*percutaneous transluminal angioplasty balloon/ or *percutaneous transluminal angioplasty/ or *angioplasty	72,918
#3	*Stent/ or *revascularization	149,863
#4	*mortality	812,936
#5	*amputation/ or major amputation.mp. or *leg amputation	47,732
#6	*Ulcer healing or *wound healing	132,836
#7	*Survival	770,209
**#8**	#1 AND (#2 OR #3) AND (#4 OR #5 OR #6 OR #7) published between January 2006 and April 2017	901

#### Predictive Factors in Ulcer Healing

Number of studies reporting predictive factors is limited [22, 28] with different predictive factors (see Table 10).

#### Predictive Factors in AFS or Limb Salvage

Predictive factors for AFS or limb salvage were reported in nine studies [21, 23–30]. All studies had different univariate and multivariate outcomes; however, age and diabetes were found to be significant predictors in at least three studies [21, 23, 24, 27]. See details in Table 10.

#### Predictive Factors in Survival Analysis

Also for the survival analysis, different predictive factors were found; however, age was found in 2 [22, 25] out of 5 studies reporting on predictive factors (see Table 10). Based on these findings, age and diabetes should be at least taken into account when searching for predictive factors.

## Discussion

### Summary

In this review, we summarized the findings on predictive factors for wound healing, AFS and survival in CLI patients who underwent a PTA. As stated, the data were heterogeneously reported and presented. In addition, none of the studies found the same predictive factors. However, in several studies age and diabetes were found as predictive factors for AFS or limb salvage and survival. Several univariate studies showed age and diabetes as predictors [12, 31–33].

### Compared with Other Studies

To our knowledge, no such systematic review has been published. There is a review [34] in which the authors summarized risk stratification models for CLI with a summary of the respective strengths and limitations of each. These models were developed from prospective cohorts to identify and quantify variables that can subsequently predict outcome in individual patients. In the prospective cohort, treatment options generally were compared (e.g., open and endovascular therapies) and new therapeutics were evaluated. The outcomes were not specific for defining risk models in patients with CLI patients undergoing PTA.

### Strength of this Review

The major strength of our study is that we focussed on patients with CLI who underwent PTA to identify possible predictive factors for clinically relevant outcomes. We have done this to create a homogeneous and clinically relevant population, in order to draw conclusions.

We included studies which aimed to study predictive values of all types of risk factors. In addition, we only selected prospective studies or studies that used a prospective database, to have a predefined design without missing a lot of data. It is known that missing data are much more common in retrospective studies, in which routinely collected data are subsequently used for a different purpose [35].

### Limitations of this Review

Although all studies were performed prospectively or a prospective database was present with a spectrum of patients which are represented, the data were presented too heterogeneously. Even the AFS or survival analysis was not reported homogeneously. The presented data on the predictive values varied even more, making general conclusions difficult.

### Conclusion and Recommendations

It is not clear which risk factors should be taken into account. However, in several studies two factors, age and diabetes, were found as predictive factors for AFS or limb salvage and survival in patients with CLI undergoing PTA. Therefore, we believe that these factors should be taken into account in the future when searching for predictive factors and when analyzing study data on endovascular treatments for CLI. More research on this topic is needed. A trial with registry of all risk factors and the outcomes up to 12 months would be very important. Future research is needed to simplify and improve the accuracy and generalizability of risk stratification in CLI.

## Appendix 1

See Table 11.
